# Urinary Biomarkers and Their Role in the Management of Urothelial Carcinoma: A Narrative Review

**DOI:** 10.3390/jcm15135183

**Published:** 2026-07-02

**Authors:** Bogdan-Petru Tichil, Anamaria Besleaga, Mihaela Laura Vica Matei, Adrian Florea

**Affiliations:** 1 Department of Cell and Molecular Biology, Faculty of Medicine, University of Medicine and Pharmacy “Iuliu Hatieganu”, 400349 Cluj-Napoca, Romania; mvica@umfcluj.ro (M.L.V.M.); adrian_a_florea@yahoo.com (A.F.); 2 Deva Emergency County Hospital, 330004 Deva, Romania; besleaga.anamaria@elearn.umfcluj.ro; 3 Department of Otorhinolaryngology, University of Medicine and Pharmacy “Iuliu Hatieganu”, 400349 Cluj-Napoca, Romania

**Keywords:** urothelial carcinoma, bladder cancer, non-muscle-invasive bladder cancer, urinary biomarkers, Xpert Bladder Cancer Monitor, Bladder EpiCheck, ADXBLADDER, Cxbladder, methylation, surveillance

## Abstract

**Background**: Urothelial carcinoma requires frequent surveillance because of its high recurrence rate, particularly in patients with non-muscle-invasive disease. Although cystoscopy remains the standard method for diagnosis and follow-up, it is invasive, costly, and associated with patient discomfort. Urinary biomarkers have emerged as potential tools for improving surveillance and reducing unnecessary cystoscopies. **Methods**: We performed a narrative review of studies published between 2017 and 2026 evaluating urinary biomarkers in urothelial carcinoma. Particular attention was given to assay mechanisms, diagnostic performance, clinical applications, and integration into surveillance techniques. **Results**: The most extensively studied biomarkers were Xpert Bladder Cancer Monitor, Bladder EpiCheck, ADXBLADDER, and Cxbladder. Most molecular assays demonstrated higher sensitivity than urinary cytology, particularly for the detection of high-grade recurrence. Reported negative predictive values frequently exceeded 95%, suggesting potential utility in identifying patients at low risk of clinically significant recurrence. Xpert Bladder Cancer Monitor and Bladder EpiCheck were supported by the largest body of surveillance evidence, whereas Cxbladder and mutation-enhanced platforms showed promise for risk stratification and individualized follow-up. Evidence supports the use of urinary biomarkers as adjuncts to cystoscopy rather than replacements. **Conclusions**: Modern urinary biomarkers provide clinically useful information during the surveillance of urothelial carcinoma, especially for excluding high-grade recurrence and assisting the interpretation of equivocal findings. Future biomarker-guided surveillance strategies may reduce the burden of cystoscopy while maintaining oncological safety. Further studies are required to improve specificity and sensitivity in order to fully integrate these biomarkers into diagnostic and follow-up protocols.

## 1. Introduction

Urothelial carcinoma represents one of the most clinically demanding malignancies encountered in urological practice, not because of uniformly aggressive biology but rather due to its unique natural history characterized by frequent recurrence and the requirement for prolonged, often lifelong surveillance. Approximately 70–75% of patients present with non-muscle-invasive bladder cancer (NMIBC), a disease entity associated with recurrence rates that may exceed 50% depending on risk stratification, thereby necessitating repeated diagnostic evaluations over extended periods [1,2,3,4].

Despite significant technological advances in oncology, white-light cystoscopy remains the diagnostic and surveillance gold standard, largely due to its ability to directly visualize bladder mucosa and enable immediate intervention [4]. However, this approach is not without limitations. It is inherently invasive; is associated with patient discomfort, anxiety, and cumulative healthcare costs; and may fail to detect flat lesions or upper tract disease [4]. In parallel, urinary cytology—although highly specific for high-grade tumors—continues to suffer from low sensitivity in low-grade disease and significant interobserver variability, thereby limiting its role as a standalone diagnostic tool [4].

These shortcomings have driven an intense search for noninvasive urinary biomarkers capable of complementing or partially replacing cystoscopy, particularly in surveillance settings. Urine, as a diagnostic medium, is uniquely suited for this purpose, given its direct contact with urothelial tissue and its content of exfoliated tumor cells, nucleic acids, proteins, metabolites, and extracellular vesicles. Tumor-derived components may enter the urine through direct shedding, active secretion, or filtration of circulating tumor-derived molecules, forming the biological basis of urine-based liquid biopsy approaches [1].

Over the past three decades, the field has evolved from simple protein-based assays such as BTA and NMP22 to increasingly sophisticated molecular platforms incorporating gene expression profiling, DNA methylation signatures, mutational analysis (e.g., FGFR3, TERT), and multi-omics integration enhanced by artificial intelligence algorithms. While early-generation biomarkers improved sensitivity, their specificity limitations prevented the widespread replacement of cystoscopy. Contemporary assays, however, have demonstrated significantly improved diagnostic performance, particularly in the detection of clinically significant, high-grade disease.

**Current Guideline Recommendations**

Major international guidelines, including those from the European Association of Urology (EAU), the National Comprehensive Cancer Network (NCCN), and the American Urological Association/Society of Urologic Oncology (AUA/SUO), recognize the increasing availability and diagnostic performance of urinary biomarkers in bladder cancer. However, all continue to recommend cystoscopy as the cornerstone of diagnosis and surveillance, with urinary biomarkers serving primarily as adjunctive tools rather than replacements for endoscopic evaluation [1,2,3].

European Association of Urology (EAU) Guidelines

The EAU Non-Muscle-Invasive Bladder Cancer (NMIBC) Guidelines acknowledge that several contemporary urinary biomarkers, including mRNA-based and DNA methylation assays, demonstrate higher sensitivity than urinary cytology, particularly for detecting high-grade recurrence. Nevertheless, the EAU concludes that current evidence is insufficient to support the routine replacement of cystoscopy during surveillance. Urinary biomarkers may be considered in selected clinical scenarios, such as clarifying equivocal cytology results, identifying patients at increased risk of recurrence, or potentially supporting risk-adapted surveillance strategies [1]. However, cystoscopic evaluation remains mandatory for the diagnosis and follow-up of NMIBC patients.

The EAU Muscle-Invasive and Metastatic Bladder Cancer (MIBC) Guidelines place less emphasis on urinary biomarkers because diagnosis and management are primarily based on histopathological confirmation, imaging, and clinical staging. At present, urinary biomarkers are not recommended for the routine diagnosis, staging, treatment selection, or surveillance of MIBC outside research settings. Emerging liquid biopsy approaches, including circulating tumor DNA (ctDNA) and urinary tumor DNA assays, are recognized as promising investigational tools but have not yet been incorporated into standard clinical practice.

National Comprehensive Cancer Network (NCCN) Guidelines

The NCCN Bladder Cancer Guidelines similarly maintain cystoscopy and urine cytology as the standard methods for bladder cancer surveillance. The NCCN recognizes that several FDA-approved urinary biomarker tests may improve the detection of recurrent disease and may provide additional information in selected patients [2]. However, the guidelines emphasize that no currently available urinary biomarker has demonstrated sufficient accuracy to replace cystoscopy.

According to the NCCN, urinary biomarkers may be considered adjuncts in situations such as equivocal cytology, the assessment of recurrence risk, or the evaluation of patients in whom cystoscopic findings are uncertain. The NCCN does not recommend the routine use of urinary biomarkers for population screening or as standalone surveillance tests. For patients with muscle-invasive disease, management decisions continue to rely on pathological staging, imaging, and multidisciplinary evaluation rather than urinary biomarker testing.

American Urological Association (AUA/SUO) Guidelines

The AUA/SUO Guideline for Non-Muscle-Invasive Bladder Cancer states that urinary biomarkers should not replace cystoscopic evaluation during surveillance. The guideline acknowledges that several biomarkers demonstrate improved sensitivity compared with urinary cytology, particularly for high-grade tumors, but notes that limitations in specificity and variability among studies prevent their routine use as substitutes for cystoscopy.

The AUA/SUO recommends the consideration of urinary biomarkers in selected circumstances, including the evaluation of patients with equivocal urine cytology, assessment of response to intravesical therapy, and surveillance of carefully selected patients when additional diagnostic information may influence management. The guideline also recognizes the potential future role of biomarkers in risk-adapted surveillance strategies but concludes that further prospective validation is required before widespread implementation [1].

Overall Guideline Consensus

Despite differences in emphasis, the EAU, NCCN, and AUA/SUO guidelines are largely consistent in their recommendations. All acknowledge that modern urinary biomarkers often provide greater sensitivity than urinary cytology, particularly for high-grade disease, and may offer clinically useful information in selected situations. However, none currently endorse urinary biomarkers as replacements for cystoscopy. Instead, contemporary guidelines position these tests as complementary tools that may assist clinical decision-making, improve risk stratification, and potentially contribute to future cystoscopy-sparing surveillance pathways once stronger evidence becomes available [1,2,3].

The aim of this study is to define the current available urinary biomarker tests and describe their utility, specificity and cost.

Unlike previous reviews that focused primarily on diagnostic accuracy, this review compares currently available urinary biomarkers according to their biological mechanisms, diagnostic performance, clinical utility, guideline recommendations, and potential role in cystoscopy-sparing surveillance pathways. In addition, recently published evidence from 2024 to 2026, including emerging data on biomarker-guided surveillance trials, is incorporated to provide a contemporary clinical perspective.

**Available Urinary Biomarkers and Biological Principles**

Urinary biomarkers can be broadly divided into cellular biomarkers and soluble biomarkers. Cellular biomarkers evaluate exfoliated tumor cells or cellular abnormalities present in urine. Examples include urinary cytology, fluorescence in situ hybridization (FISH), and ImmunoCyt. Soluble biomarkers evaluate proteins, messenger RNA, DNA mutations, DNA methylation patterns, extracellular vesicles, or cell-free nucleic acids released by tumor cells into urine [4].

Protein-based biomarkers include NMP22, bladder tumor antigen (BTA), and MCM5 (ADXBLADDER test). These tests detect proteins released by urothelial tumor cells but may be influenced by inflammation, infection, or hematuria [4].

Messenger RNA-based biomarkers, included in the Xpert Bladder Cancer Monitor and Cxbladder, detect altered gene expression patterns associated with urothelial carcinoma. Their principal advantage is increased sensitivity for recurrent disease, particularly high-grade recurrence [5,6,7,8,9,10].

DNA methylation assays, represented primarily by Bladder EpiCheck, identify epigenetic alterations associated with malignant transformation and are particularly useful in patients with atypical cytology [11,12,13,14,15].

Mutation-based platforms evaluate genomic alterations such as FGFR3 and TERT promoter mutations. These alterations can improve risk stratification when combined with transcriptomic assays [8].

Emerging liquid biopsy approaches include cell-free DNA, circulating tumor DNA, exosomal RNA, and multi-omics platforms integrating genomic, transcriptomic, proteomic, and metabolomic information [16,17,18].

## 2. Materials and Methods

### 2.1. Review Design

This article represents a narrative review of the available literature from January 2017 to March 2026. The corpus of literature studied includes original cohort studies, comparative diagnostic studies, translational case-based work, narrative reviews, systematic reviews, a meta-analysis, and a clinical trial protocol.

### 2.2. Scope of Evidence

The included literature addressed the following biomarker domains:

1. mRNA assays, especially Xpert Bladder Cancer Monitor and Cxbladder.
2. DNA methylation assays, especially Bladder EpiCheck and related methylation-based recurrence studies.
3. Protein marker review evidence, especially ADXBLADDER.
4. Mutation-augmented platforms, especially FGFR3- and TERT-enhanced Cxbladder.
5. Broader liquid biopsy concepts, especially ctDNA.
6. Clinical scenarios, including NMIBC surveillance, primary hematuria, atypical cytology, active surveillance, second-TURB prediction, and upper tract urothelial carcinoma assessment.

### 2.3. Outcomes of Interest

The primary outcomes extracted conceptually from the studies used were sensitivity, specificity, PPV, NPV, high-grade detection, recurrence prediction, and clinical utility with respect to potential cystoscopy reduction. Secondary outcomes included performance in atypical cytology, upper tract applications, mixed diagnostic/follow-up populations, and the impact of biomarker integration on clinical decision-making.

## 3. Results

### 3.1. The Broader Biomarker Landscape and the Clinical Need for Noninvasive Surveillance

The earliest broad review in the reviewed literature, by Miyake et al., framed the problem clearly: urothelial carcinoma is not difficult simply because it is common but because current monitoring modalities are simultaneously indispensable and unsatisfactory [19]. This foundational framing remains strikingly compatible with the newer literature and helps explain why contemporary biomarker work has expanded so quickly [5].

The 2022 hematuria meta-analysis also reinforces the same clinical premise from a diagnostic rather than surveillance perspective [20]. The authors evaluated FDA-approved biomarkers in primary hematuria and found that, although some tests such as Cxbladder and AssureMDx performed better than others, current diagnostic performance remained insufficient for broad adoption as universal rule-out tools or triage substitutes for cystoscopy [20]. This distinction is critical: biomarker performance can be promising and still not yet practice-changing in all settings.

One of the most consistent insights across the urinary biomarker landscape is that surveillance and primary diagnosis should not be conflated. In surveillance populations, where pretest probabilities and clinical context are known, a biomarker with strong high-grade NPV may be extraordinarily useful. In primary hematuria, where the diagnostic field is wider and the acceptable miss rate lower, the evidentiary threshold for cystoscopy replacement is much higher [20,21,22,23,24,25,26,27,28,29,30,31].

### 3.2. Xpert Bladder Cancer Monitor: The Dominant mRNA Surveillance Assay

One of the most used urinary biomarker tests currently is the Xpert Bladder Cancer Monitor, making it possible to observe its performance across conventional surveillance, active surveillance, procedural triage, and exploratory upper tract settings. Across these studies, the broad pattern is highly reproducible: Xpert repeatedly outperforms cytology in sensitivity, especially for high-grade recurrence, but sacrifices specificity [5,23,24,25,26,27,28,29].

#### 3.2.1. Large Surveillance Cohorts

The prospective update by D’Elia et al. included 1015 samples from 416 NMIBC follow-up patients and identified 168 recurrences, 75% of which were low-grade and 25% high-grade. Overall sensitivity was 17.9% for cytology and 52.4% for Xpert [5]. Cytology detected only 6.3% of low-grade recurrences, whereas Xpert detected 42.9%; in terms of high-grade recurrence, cytology detected 52.4%, while Xpert detected 80.9% [5]. This study is particularly useful because it clearly demonstrates that the added value of Xpert is not marginal; it is clinically substantial, especially where high-grade recurrence is concerned.

Cancel-Tassin et al. reported equally compelling findings in a French prospective follow-up cohort of 500 NMIBC patients [27]. Overall sensitivity, specificity, and NPV were 72.7%, 73.7%, and 96.5% for Xpert, compared with 7.7%, 97.8%, and 92.8% for cytology [27]. Xpert detected 92.3% of high-grade tumors and ruled them out with an NPV of 99.7% [27]. For any clinician considering whether a biomarker may safely support less invasive follow-up, a high-grade NPV of 99.7% is among the most persuasive numerical findings in the entire studied literature.

Cowan et al. added a large multicenter longitudinal perspective [28]. In 429 surveillance patients, histology-confirmed recurrence occurred in 13.5%. Xpert showed an overall sensitivity of 60.3% and specificity of 76.5%, with a sensitivity of 87% and NPV of 99% for high-grade recurrence. Particularly important was the observation that patients with positive Xpert but negative cystoscopy were at much higher subsequent recurrence risk than double-negative patients, suggesting that at least part of the biomarker’s apparent false-positive signal may represent biologically meaningful early warning [28].

Smrkolj et al. reached a similar conclusion in a smaller but methodologically straightforward comparison with voided urinary cytology [29]. For malignant histopathology, Xpert achieved a sensitivity of 76.9%, specificity of 97.5%, and NPV of 93.0%, versus 38.4%, 97.5%, and 83.3% for cytology. Xpert achieved 100% sensitivity for high-grade tumors in that series, compared with 42.9% for cytology [29]. The authors proposed that Xpert could increase follow-up cystoscopy intervals and interestingly found that combining it with cytology added little beyond Xpert alone [29].

#### 3.2.2. Xpert in Strategy-Level and Special Use Studies

Singer et al. evaluated Xpert alongside narrow-band imaging cystoscopy in a prospective, double-blinded monocentric study [25]. Their results suggested that narrow-band imaging provided no meaningful benefit over conventional white-light cystoscopy, whereas Xpert performed especially well in aggressive high-grade recurrence [25]. The study therefore subtly shifts the discussion from “better cystoscopy optics” toward “better biology-driven surveillance” [25].

Lozano et al. asked the most direct clinical question regarding whether cystoscopy could be substituted [26]. In 337 patients followed within the first two years after NMIBC diagnosis, Xpert had a sensitivity of 69.4% for any recurrence and 63.6% for high-risk recurrence, with an NPV of 93% and 96.2%, respectively [26]. Although the assay could theoretically have avoided many invasive controls, it would also have missed a clinically meaningful number of recurrences, including high-risk lesions. The authors therefore concluded that Xpert was not sufficiently sensitive to replace cystoscopy outright. Yet they also showed that false-positive Xpert results predicted early future recurrence and that threshold optimization could improve the detection of high-risk disease. This study is therefore best read not as a negative trial but as evidence that the biomarker is clinically useful when intelligently calibrated, rather than when simplistically used as a binary replacement test [26].

Fasulo et al. moved the conversation into the active surveillance of recurrent NMIBC [24]. In that setting, sequential negative Xpert tests suggested that a large proportion of unnecessary cystoscopies might be avoided, especially after repeated negative results [24]. This is conceptually important because serial negative molecular testing may have greater clinical utility than isolated one-time negativity.

Breyer et al. used Xpert for a different purpose entirely: the prediction of second-TURB necessity after initial resection [23]. Although clinical assessment outperformed the assay overall, Xpert achieved an NPV of 92.1% overall and 96% in pTa disease, suggesting that the biomarker-guided omission of repeat resection may become conceivable in carefully selected settings [6]. This expands the possible relevance of Xpert beyond surveillance into procedural triage.

#### 3.2.3. Xpert Beyond Bladder-Only Surveillance

D’Elia et al. explored Xpert Detection in suspected UTUC and suggested that upper tract urine testing may have diagnostic potential, though their study was explicitly preliminary [22]. Kavcic et al. likewise showed that Xpert Detection, used alongside UroVysion, detected a subset of urothelial carcinomas that conventional evaluation might otherwise have missed in a mixed cohort of hematuria and post-treatment patients [13]. These studies remain exploratory, but they imply that Xpert-like assays may eventually contribute to a broader pan-urothelial monitoring paradigm.

### 3.3. Bladder EpiCheck and the Maturation of Methylation-Based Biomarkers

Bladder EpiCheck is the principal methylation-based platform and represents a different biological philosophy from mRNA assays. Instead of focusing on transcriptional overexpression, it detects altered DNA methylation patterns associated with urothelial carcinoma [13,14]. This may offer advantages in settings where cytologic morphology is compromised or equivocal.

D’Andrea et al. provided one of the strongest EpiCheck studies from a clinical utility standpoint [11]. They showed that the test improved recurrence prediction when added to standard clinicopathologic variables and that decision curve analysis suggested reduced unnecessary investigations across a wide threshold range. This is particularly important because it moves beyond static diagnostic statistics and examines whether the biomarker can change clinical decisions in a useful way.

Peña et al. evaluated EpiCheck specifically in follow-up patients whose cytology showed atypical urothelial cells [12]. This population is clinically frustrating because atypia frequently leads to repeated testing without clear resolution. Their study concluded that the urine methylation test was useful in both follow-up and the diagnostic clarification of atypia [12]. In practice, this may be one of the most valuable uses of methylation testing because it addresses a scenario in which conventional morphology is the least decisive.

The reviews by Mancini et al. and Fiorentino et al. strengthen this broader interpretation [13,14]. They summarize evidence showing that EpiCheck generally provides sensitivity and NPV superior to cytology, particularly for high-grade lesions, although cytology retains better specificity [13,14]. Fiorentino et al. also discuss possible use in upper tract disease and emphasize that combining cytology with methylation analysis may improve diagnosis and recurrence prediction in uncertain cases [14].

Pierconti et al. extended the methylation story into recurrence risk prediction, proposing that methylation analysis in urinary samples may help identify high-risk patients more likely to recur [15]. Their later paper on invalid results is also noteworthy because it reflects the maturation of the field from proof of concept toward implementation-level concerns [15].

### 3.4. Direct Comparison of Xpert, EpiCheck, and Cytology

Trenti et al. provided one of the most practically useful studies in the uploaded corpus by directly comparing the two leading contemporary urinary markers with cytology in NMIBC follow-up [30]. Overall sensitivity was 27.17% for cytology, 64.13% for EpiCheck, and 66.3% for Xpert [18]. Specificity was 98.82% for cytology, 82.06% for EpiCheck, and 76.47% for Xpert [18]. When the two molecular tests were used together, overall tumor detection increased to 79.35%, including 92.11% of high-grade tumors [30].

This single study illustrates the balance between higher sensitivity and lower specificity that characterizes most contemporary urinary biomarkers better than almost any review: cytology remains extremely specific, but molecular assays dramatically improve sensitivity and high-grade capture [30]. It also suggests that molecular complementarity may matter; the future may not belong to one “winner” biomarker but to integrated strategies combining complementary biological signals [30].

### 3.5. ADXBLADDER and the Protein Biomarker Perspective

ADXBLADDER is represented chiefly through the detailed review by Wolfs et al. [31]. This review is important because it places ADXBLADDER and EpiCheck side by side as newer-generation assays with stronger surveillance promise than many earlier urine markers [31]. Across the reviewed prospective studies, ADXBLADDER showed a sensitivity of 45–73%, an NPV of 74–100%, and particularly favorable performance for high-grade recurrence, where sensitivity rose to 76–88% and NPV approached 99% [31].

Even though ADXBLADDER is less heavily represented by primary uploaded studies than Xpert or EpiCheck, its inclusion is still informative because it broadens the conclusion that modern non-cytology biomarkers tend to share a common value proposition: better sensitivity and better high-grade exclusion than cytology but lower specificity and continued dependence on contextual clinical interpretation [31].

### 3.6. Cxbladder, Mutation-Augmented Platforms, and Pathway Redesign

The literature concerning Cxbladder contributes a somewhat different perspective because it emphasizes clinical utility and decision pathway redesign. Earlier studies showed that when clinicians were given Cxbladder results, they altered their use of invasive procedures and reduced unnecessary testing while still identifying clinically relevant cancers [7,31]. Real-world surveillance analyses suggested that selected low-risk patients could safely undergo fewer cystoscopies, and a pandemic-era study found that Cxbladder Monitor allowed some patients to skip scheduled surveillance cystoscopy without adverse short-term oncologic findings [9,10].

The mutation-enhanced study by Lotan et al. is especially relevant because it demonstrates the benefit of combining different forms of molecular information, such as gene expression and mutation analysis, within a single diagnostic platform [8]. By adding FGFR3 and TERT mutation analysis to the mRNA framework of Cxbladder, the authors improved specificity for the triage assay and improved both sensitivity and specificity for the detect assay, with an NPV of 99.7% for enhanced detection and a large fraction of patients potentially spared cystoscopy [8]. This is one of the clearest indications in the corpus that next-generation biomarker performance may depend less on refining a single platform and more on combining transcriptomic and genomic information.

### 3.7. Primary Hematuria, Upper Tract Disease, and Difficult Diagnostic Scenarios

The hematuria meta-analysis by Soputro et al. is critical because it prevents the overextension of surveillance-derived conclusions into de novo diagnostic work-up [20]. Although Cxbladder and some other assays performed comparatively well, the authors still concluded that current biomarkers remain insufficient for general use as universal rule-out tools or cystoscopy triage tests in primary hematuria [20]. This means that biomarker enthusiasm must remain calibrated to context. What is plausible in established NMIBC surveillance is not automatically transferable to all hematuria evaluations.

Upper tract disease remains a promising but relatively early application domain [13,14,22]. Xpert Detection in UTUC suspicion, upper tract discussions within the EpiCheck review, and mixed diagnostic/follow-up genetic test validation all suggest feasibility, but none of the examined studies are strong enough to support routine upper tract adoption at present.

The case-based report by Kałuzewski et al. deserves mention because it demonstrates how multiple urinary modalities—cytology, immunocytochemistry, FISH, EpiCheck, and whole-genome sequencing—can converge in a difficult real-world case to clarify diagnosis and management [32]. It also illustrates the growing overlap between biomarker diagnostics and actionable molecular oncology.

### 3.8. Future Directions: ctDNA and Randomized Biomarker-Guided Surveillance

Christensen et al. reviewed ctDNA in bladder cancer and argued that circulating tumor DNA is likely to become increasingly relevant in disease monitoring and clinical trial design [33]. Although ctDNA is not yet a practical replacement for urine-based surveillance in NMIBC, the future of urothelial cancer follow-up may involve integrated urine and blood molecular surveillance rather than dependence on a single test modality.

The most concrete evidence that the field is advancing toward practice change is the 2024 “Replace Cysto” protocol [34]. This randomized phase 2 trial compares alternating urine marker surveillance with Xpert or EpiCheck against frequent scheduled cystoscopy in low-grade intermediate-risk NMIBC [34]. Its primary endpoint is urinary quality of life, and its exploratory outcomes include invasive procedure burden, complications, recurrence, and progression [34]. This is a major conceptual step: the field is no longer asking only whether biomarkers can detect disease but whether they can safely improve the patient experience of surveillance itself.

### 3.9. Multi-Omics Integration and the Evolution Toward Precision Liquid Biopsy

One of the most significant conceptual advancements in the field of urinary biomarkers is the transition from single-analyte detection to multi-omics integration, a paradigm that combines genomic, transcriptomic, proteomic, and metabolomic data to improve diagnostic performance and biological insight [17].

Urine represents an exceptionally rich biological substrate for such approaches, containing:

- Cell-free DNA (cfDNA).
- mRNA and noncoding RNA (miRNA, lncRNA).
- Tumor-derived proteins.
- Metabolic signatures.
- Extracellular vesicles (exosomes).

These components originate from multiple mechanisms, including direct tumor shedding, apoptosis-mediated release into circulation followed by renal filtration, and active secretion by tumor cells, thereby providing a comprehensive molecular snapshot of tumor biology [17].

Recent technological developments include:

- Next-generation sequencing (NGS).
- Mass spectrometry-based metabolomics.
- Digital PCR.
- AI-assisted pattern recognition.

These have significantly enhanced both analytical sensitivity and specificity, enabling earlier detection and more accurate disease monitoring [17,34].

Importantly, ideal urinary biomarkers must satisfy several stringent criteria:

1. Tumor specificity.
2. Stability independent of physiological variation.
3. Detectability at early disease stages.
4. Reproducibility across platforms and populations.

However, despite these advances, several challenges remain:

- Lack of standardization in sample collection and processing.
- Inter-individual biological variability.
- High cost of advanced detection technologies.
- Need for large-scale prospective validation studies.

These limitations underscore that, while multi-omics approaches represent the future of urothelial carcinoma diagnostics, their clinical translation remains ongoing rather than complete [17].

**Cost and Practical Considerations**

The adoption of urinary biomarkers depends not only on diagnostic performance but also on cost, availability, laboratory infrastructure, reimbursement policies, and their potential to reduce the frequency of cystoscopy during surveillance. Although exact prices vary according to country, healthcare system, and contractual agreements, the approximate costs reported in the United States and Europe provide a useful comparison of currently available tests. Importantly, cost-effectiveness is closely linked to diagnostic accuracy, particularly sensitivity, specificity, and negative predictive value (NPV), as tests with high specificity reduce unnecessary investigations, while those with high NPV may safely decrease cystoscopy burden in selected patients [18,35].

Conventional urinary cytology remains the least expensive and most widely available test, with a typical cost of approximately USD 50–150 (€45–140) per sample. Cytology demonstrates excellent specificity, often exceeding 90–95%, particularly for high-grade urothelial carcinoma, but its sensitivity for low-grade tumors is poor, ranging from 10 to 40% [36,37]. Consequently, despite its low cost and high specificity, cytology alone is insufficient as a replacement for cystoscopy.

Among older FDA-approved protein-based assays, NMP22 generally costs approximately USD 25–100 (€20–90) per test and demonstrates pooled sensitivities of approximately 56–69% and specificities of 77–88% [38,39]. BTA Stat and BTA TRAK, costing approximately USD 30–120 (€25–110), show sensitivities ranging from 57 to 83% but lower specificities (68–85%) because false-positive results frequently occur in patients with hematuria, urinary tract infection, inflammation, or recent instrumentation [38,40]. Although relatively inexpensive, their reduced specificity limits their clinical utility and may increase downstream costs through unnecessary cystoscopic evaluations.

ADXBLADDER, which detects the minichromosome maintenance protein MCM5 released by proliferating urothelial tumor cells, generally costs approximately USD 100–200 (€90–180) per test. Reported sensitivities range from 51 to 73%, with specificities of approximately 66–73%, while sensitivity for high-grade recurrence may exceed that of cytology [41,42]. Its relatively simple laboratory workflow and improved detection of clinically significant disease support its use as an adjunctive surveillance tool.

The mRNA-based Xpert Bladder Cancer Monitor assay typically costs approximately USD 150–300 (€140–280) per test. The assay provides rapid automated analysis and has demonstrated sensitivities of 60–90%, specificities of 75–91%, and NPVs frequently exceeding 95% for high-grade recurrence [11,43]. These characteristics make it particularly attractive for surveillance strategies aimed at reducing unnecessary cystoscopies while maintaining patient safety.

The DNA methylation assay Bladder EpiCheck generally costs approximately USD 200–350 (€180–320) per test. Published studies report sensitivities of 62–90%, specificities of 82–88%, and NPVs above 94–97% for high-grade disease [44]. Although more expensive than cytology, its high specificity and excellent NPV may improve cost-effectiveness by reducing invasive follow-up procedures, particularly in patients with atypical or equivocal cytology.

Cxbladder, a multigene mRNA-based platform, is among the most expensive commercially available urinary biomarker tests, with costs typically ranging from USD 350 to 800 (€320–750) depending on the specific assay and healthcare setting. Diagnostic performance varies according to the assay version, but reported sensitivities often exceed 90%, with NPVs approaching 95–98%, while specificity generally ranges from 60 to 85% [45,46,47]. Several studies suggest that its high sensitivity may reduce the number of unnecessary cystoscopies, potentially offsetting part of its initial cost through decreased procedural utilization.

UroVysion FISH, which evaluates chromosomal abnormalities in exfoliated urothelial cells, generally costs approximately USD 500–1000 (€450–900) per test and requires specialized laboratory expertise. Reported sensitivities range from 61 to 72%, with specificities of 78–89% [48,49]. Although more sensitive than cytology for some recurrent tumors, its high cost and technical complexity limit widespread routine use.

Emerging technologies based on circulating tumor DNA (ctDNA), urinary tumor DNA, next-generation sequencing (NGS) panels, and multi-omics platforms currently have the highest costs, frequently exceeding USD 500–2000 (€450–1850) per analysis. Early studies have reported sensitivities and specificities frequently above 80–90%, particularly when multiple genomic and epigenomic markers are combined [50,51]. However, these technologies remain largely confined to research settings and specialized centers, and robust prospective validation is still required before routine clinical implementation.

From a health economic perspective, the value of urinary biomarkers depends not only on the direct cost of testing but also on their ability to reduce cystoscopies, outpatient visits, procedure-related complications, and healthcare utilization. Biomarkers with high specificity can decrease false-positive findings and avoid unnecessary invasive procedures, whereas those with high NPVs may support risk-adapted surveillance protocols that safely reduce cystoscopy frequency [35,36]. Current evidence suggests that the greatest economic benefit may be achieved in carefully selected surveillance populations [Table 1], particularly patients with low- and intermediate-risk non-muscle-invasive bladder cancer. Nevertheless, further prospective cost-effectiveness studies are required before widespread implementation can be recommended.

## 4. Discussion

The examined literature supports a far more mature view of urinary biomarkers than was possible even a few years ago. The collective evidence now shows that modern urine-based assays are not marginal adjuncts but meaningful clinical tools, especially in NMIBC surveillance [5,10,11,14,30,31]. Xpert, EpiCheck, ADXBLADDER, and Cxbladder-derived platforms all contribute to the same broad conclusion: contemporary biomarkers frequently outperform cytology in sensitivity and often achieve very high NPV for high-grade recurrence, the endpoint of the greatest practical importance in cystoscopy-sparing strategies.

At the same time, the evidence does not support simplistic claims that cystoscopy can now be abandoned. The main limitation remains specificity. Cytology is still generally more specific than molecular assays, and positive biomarker results in the face of negative endoscopy can create uncertainty [20,26,30]. Yet the longitudinal Xpert data suggest that some of these discordant results are biologically meaningful, which complicates the interpretation of a so-called false positive [26,28]. This may be especially important in future algorithm design, where a positive marker plus negative cystoscopy might trigger intensified follow-up rather than immediate labeling as assay error.

Another major lesson from the corpus is that biomarkers are increasingly valuable as decision tools rather than merely detection tools. D’Andrea et al. used decision curve analysis for EpiCheck [11]. Fasulo et al. evaluated serial Xpert negativity in active surveillance [24]. Breyer et al. tested Xpert as a second-TURB predictor [23]. Cxbladder studies demonstrated changes in physician behavior and real-world cystoscopy reduction [6,7,9,10]. The “Replace Cysto” protocol formalizes this evolution by directly randomizing patients to biomarker-guided surveillance strategies [33]. Together, these studies show that the central question is shifting from “Does the biomarker detect recurrence?” to “Can the biomarker safely change what clinicians do?”.

The strongest immediate clinical role of urinary biomarkers therefore appears to lie in four areas:

1. High-grade recurrence exclusion.
2. Interpretation of atypical or equivocal findings.
3. Longitudinal surveillance de-intensification in selected patients.
4. Integration into multivariable or multimodal risk-adapted pathways [8,11,12,30,33].

The limitations of this manuscript are chiefly methodological rather than conceptual. It is a comprehensive synthesis of the studied literature, not a de novo unrestricted systematic review of all the published literature. The underlying studies are heterogeneous in terms of assay thresholds, recurrence prevalence, endpoints, and comparator standards.

## 5. Conclusions

The reviewed papers support a strong, clinically relevant conclusion: urinary biomarkers in urothelial carcinoma have moved beyond exploratory interest and now occupy a meaningful position in personalized surveillance. Their present role should not be to universally replace cystoscopy but to reduce unnecessary invasive follow-up, clarify equivocal findings, and provide high-confidence exclusion of clinically important recurrence, especially high-grade disease.

The next decisive stage for the field will be the transition from diagnostic accuracy to outcome-based surveillance redesign. At the moment, more studies are required to improve specificity and sensitivity so as to safely incorporate the tools into the standard follow-up of urothelial cancer patients.

## Figures and Tables

**Table 1:** Diagnostic performance and approximate costs of urinary biomarkers.

Test Type	Specificity (%)	Sensitivity (%)	Approximate Price Range (USD/EUR)
Urinary Cytology	90–95	10–40	USD 50–150/€45–140
NMP22	77–88	56–69	USD 25–100/€20–90
BTA Stat/BTA TRAK	68–85	57–83	USD 30–120/€25–110
ADXBLADDER (MCM5)	66–73	51–73	USD 100–200/€90–180
Xpert Bladder Cancer Monitor	75–91	60–90	USD 150–300/€140–280
Bladder EpiCheck	82–88	62–90	USD 200–350/€180–320
Cxbladder	60–85	>90	USD 350–800/€320–750
UroVysion FISH	78–89	61–72	USD 500–1000/€450–900
ctDNA/Urinary Tumor DNA Assays	80–90+	80–90+	USD 500–2000/€450–1850
Multi-Omics/NGS Platforms	80–90+	80–90+	USD 500–2000/€450–1850

Diagnostic performance and approximate costs of currently available urinary biomarkers for urothelial carcinoma surveillance and detection. Sensitivity and specificity ranges are derived from representative studies and meta-analyses and may vary according to patient population, disease prevalence, assay version, and clinical setting. Cost estimates represent approximate ranges reported in North American and European healthcare systems [11,18,35,36,37,38,39,40,41,42,43,44,45,46,47,48,49,50,51].

## Data Availability

No new data were created or analyzed in this study.

## Abbreviations

The following abbreviations are used in this manuscript:

AS	active surveillance
AUC	area under the curve
BC	bladder cancer
BCM	Bladder Cancer Monitor
ctDNA	circulating tumor DNA
CTU	computed tomography urography
HG	high grade
LG	low grade
LDA	linear discriminant analysis
NMIBC	non-muscle-invasive bladder cancer
NPV	negative predictive value
PPV	positive predictive value
TURB	transurethral resection of bladder tumor
URS	ureterorendoscopy
UTUC	upper tract urothelial carcinoma
VUC	voided urinary cytology
WLC	white-light cystoscopy
